# The Role of Imaging in Patient Selection, Preoperative Planning, and Postoperative Monitoring in Human Upper Extremity Allotransplantation

**DOI:** 10.1155/2014/169546

**Published:** 2014-03-27

**Authors:** Eira S. Roth, David G. Buck, Vijay S. Gorantla, Joseph E. Losee, Daniel E. Foust, Cynthia A. Britton

**Affiliations:** ^1^Department of Diagnostic Radiology, University of Pittsburgh Medical Center (UPMC), C/o Patricia O'Kelly, 200 Lothrop Street Presbyterian South Tower Suite 3950, Pittsburgh, PA 15213, USA; ^2^Department of Interventional Radiology, The Western Pennsylvania Hospital, 4800 Friendship Avenue, Pittsburgh, PA 14224, USA; ^3^Department of Plastic Surgery, University of Pittsburgh Medical Center, 3550 Terrace Street Scaife Hall, Suite 678, Pittsburgh, PA 15261, USA

## Abstract

*Objective*. To describe the role of imaging in vascular composite allotransplantation based on one institution's experience with upper extremity allotransplant patients. *Methods*. The institutional review board approved this review of HIPAA-compliant patient data without the need for individual consent. A retrospective review was performed of imaging from 2008 to 2011 on individuals undergoing upper extremity transplantation. This demonstrated that, of the 19 patients initially considered, 5 patients with a mean age of 37 underwent transplantation. Reports were correlated clinically to delineate which preoperative factors lead to patient selection versus disqualification and what concerns dictated postoperative imaging. Findings were subdivided into musculoskeletal and vascular imaging criterion. *Results*. Within the screening phase, musculoskeletal exclusion criterion included severe shoulder arthropathy, poor native bone integrity, and marked muscular atrophy. Vascular exclusion criterion included loss of sufficient arterial or venous supply and significant distortion of the native vascular architecture. Postoperative imaging was used to document healing and hardware integrity. Postsurgical angiography and ultrasound were used to monitor for endothelial proliferation or thrombosis as signs of rejection and vascular complication. *Conclusion*. Multimodality imaging is an integral component of vascular composite allotransplantation surgical planning and surveillance to maximize returning form and functionality while minimizing possible complications.

## 1. Introduction

Vascularized Composite Allotransplantation, or VCA, refers to the transfer and integration of multiple tissue components and has generally been used to describe nonorgan transplants such as face and extremity [1]. The goal of such procedures is to restore both form and functionality following catastrophic injury. The first such procedure was performed by Peacock in the form of an “en bloc digital flexor mechanism transplant” in 1957 [2–4]. However, the successful investigation into complete extremity transplantation occurred only after the introduction of cyclosporine in 1982, paving the way for the first successful unilateral hand transplant which occurred in Lyon France in September 1998 [4, 5]. Since then, there have been 22 unilateral and 23 bilateral hand transplants recordedby the International Registry of Hand and Composite Tissue Transplantation [6, 7].

Although VCA includes a range of surgical procedures such as face and extremity transplantation, this paper focuses primarily on our institutional experience with the recipients of hand allotransplantation. Hand transplantation involves the systematic integration of donor upper extremity tissues to the recipient beginning with the attachment of bone, followed by tendons, nerves, vessels, and cutaneous tissues, with multiple teams working in tandem to attach the donor limb. Given the surgical complexity, extensive presurgical planning and close follow-up are required. It is therefore imperative that clinicians be cognizant of which radiologic findings are pertinent in operative planning and subsequent patient care, particularly as VCA transplantation becomes more common. Thus, the aim of this paper is to utilize our institutional experience in order to optimize the radiologic understanding of this unique patient population, about whom little exists in the current imaging literature.

## 2. Subjects and Methods

The institutional review board approved this retrospective review of HIPAA-compliant patient data, without the need for individual consent. 150 patient referrals were reviewed which yielded 19 patients that were initially considered for upper extremity allotransplantation. Of these, five patients ranging in age between 27 and 59 years with a mean age of 37 underwent transplantation. Three males and two females were selected, with three having experienced amputation secondary to extremity gangrene from sepsis and two having undergone traumatic amputation. Three of the five patients had experienced quadrilateral amputations. This group underwent a combined total of 8 upper extremity transplantations. A systematic retrospective review was performed of the imaging and clinical records obtained from 2008 to 2011. This review included both preoperative screening and postoperative surveillance imaging within the musculoskeletal and vascular radiology subdivisions.

### 2.1. Presurgical Work-Up

Individuals considered for transplant candidacy underwent extensive preoperative radiologic evaluation; all began with conventional digital radiography of the residual limb to evaluate bone integrity and the length of the remaining long bones. However, the combination of subsequent imaging modalities was individualized based on each patient's mechanism of injury, surgical history, and initial findings on radiography.

Radiographs were obtained at the level of injury and proximally, with particular attention to the inclusion of the proximal joints. 64-slice CT (GE LightSpeed VCT) with 2D reformatting was obtained when further characterization of bone defects, such as displacement of residual bone fragments and fracture extension, was needed. Those with prior reconstruction attempts underwent 1.5 Tesla (GE HD16.0) MRI to determine the extent of healthy residual tissue, muscle bulk, and tendon integrity.

Conventional angiography or CT angiography (GE Innova flat panel Model 2329766 or Siemens Multistar TOP image intensifier model 03135584) was obtained preoperatively to characterize the residual vascular architecture, often distorted both by initial injury and subsequent surgery. Angiography was also used to identify suitable vessels for allograft anastomosis to ensure sufficient vascular supply.

Imaging was also performed to exclude subclinical systemic disease that could contraindicate the use of long-term immunosuppressive therapy. 1.5 Tesla MRI of the hips was performed on all patients to excluded preexisting avascular necrosis. Abdominal ultrasound was used to exclude subclinical abdominal pathology, and maxillofacial radiographs were requested to exclude significant sinus disease (Table 1).

### 2.2. Postsurgical Work-Up

Immediate postsurgical surveillance consisted primarily of radiography of the affected arm. This was followed by sequential follow-up radiographs at 1, 3, 9, and 12 months, and yearly, to monitor bone healing. CT and MRI were again used to describe any complication noted either radiographically or physically. Repeated angiography was also performed if clinical symptoms developed that suggested arterial or venous stenosis or thrombosis. Long-term surveillance consisted of routine peripheral vascular ultrasounds performed separately by the clinical service to assure continued patency of the anastomosis and distal vessels, attempting to monitor for endothelial proliferation as a possible marker of rejection. However, as these ultrasound examinations were performed outside of the radiology department, the images were unavailable for review.

## 3. Results

Exclusion from candidacy was based on the weighting of multiple factors, some of which were difficult to quantify retrospectively as initial consideration for qualification occurred prior to imaging evaluation. Of the subsequent 19 individuals, ten were excluded based on psychosocial criterion beyond the scope of this paper. The nine remaining underwent imaging evaluation which revealed a combination of the below findings (Table 2).

### 3.1. Musculoskeletal Presurgical Work-Up

**Figure 1:**
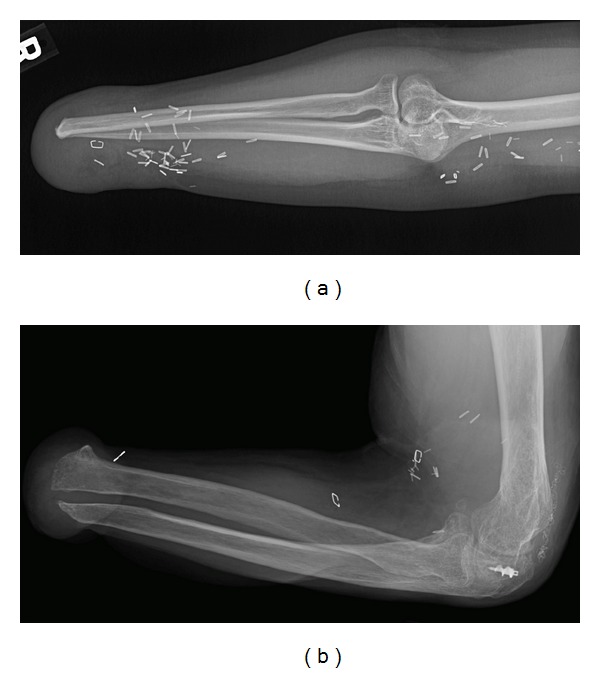
(a) Radiograph of selected candidate shows osseous integrity of the amputation site and no significant arthropathy of the proximal joint. (b) Radiograph showing diffuse osteopenia and proximal arthropathy of a patient who failed screening.

All remaining individuals underwent radiography of their injured extremities (Figure 1(a)). Findings that factored into exclusion from transplant candidacy of three individuals consisted primarily of insufficient native bone and soft tissues to support an allograft (Figure 1(b)). This occurred secondary to extremity loss with maceration of the residual limb that resulted in extensive osseous compromise, fracture, and fragmentation. Two individuals were disqualified when it was evident that prior surgical revisions had left insufficient viable soft tissue to support VCA, with one patient also demonstrating clinical evidence of prior skin graft failure. Joint arthropathy was deemed a relative contraindication depending on the severity and the location. Screened patients with severe shoulder arthropathy were not accepted, contributing to the disqualification of one person. Individuals with distal arthropathy were considered for transplantation above the level of the involved joint.

Despite multiple prior surgeries and the various causes of extremity loss, all of the individuals accepted for transplant maintained sufficient healthy bone to permit transplantation at the level of the mid forearm or mid-humerus. One of the five had degenerative disease of the wrist, prompting the decision to extend the level of the transplant to include that joint. None of the chosen individuals showed any evidence of underlying systemic disease per abdominal ultrasound or maxillofacial radiography.

**Figure 2:**
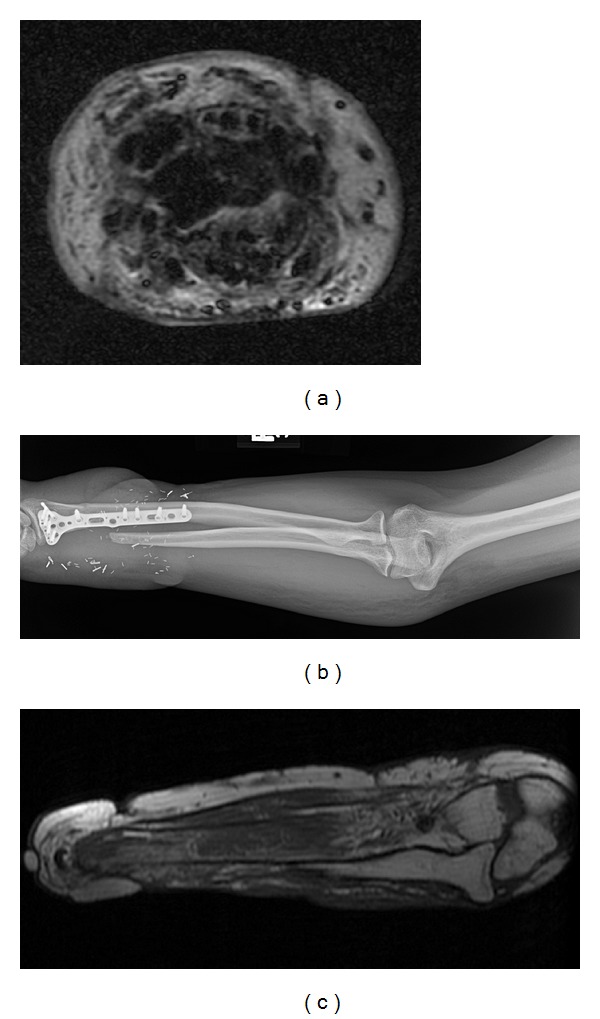
(a) Axial IR MRI without contrast of the extremity shows circumferential edema and skin thickening consistent with cellulites. This patient underwent antibiotic therapy prior to transplantation. (b) Corresponding radiograph showing extensive soft tissue edema. (c) MRI of a different patient demonstrated diffuse muscle atrophy. This patient was disqualified from consideration.

MRI was utilized on four people only to further characterize suspected pathology. One individual underwent MRI for bilateral upper extremity cellulites to exclude osteomyelitis and was accepted for transplantation following antibiotic treatment (Figure 2(a)). Another person failed screening when found to have marked muscle atrophy of the residual limb, indicating underlying irreversible denervation injury (Figure 2(b)). A third individual with a history of femoral head avascular necrosis underwent bilateral arthroplasty prior to transplant consideration. The fourth MRI demonstrated preserved muscle bulk despite limited upper extremity functionality secondary to contractures.

### 3.2. Vascular Presurgical Planning

These patients also underwent presurgical conventional angiography or CT angiography. All of the 5 individuals chosen for transplantation showed relative preservation of normal vascular anatomy approximating the residual limb with retained patency of at least one major vessel (ulnar and/or radial) to serve as the anastomotic vascular pedicle.

**Figure 3:**
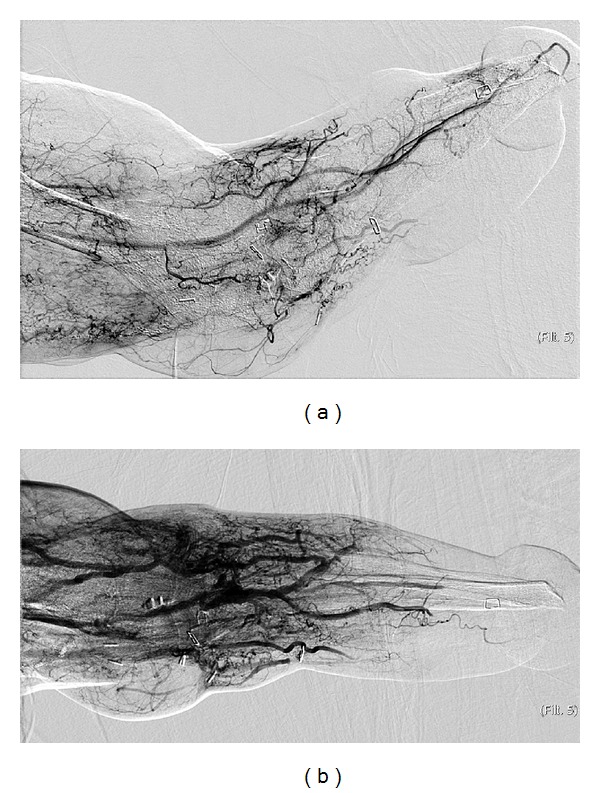
(a) Angiographic findings resulting in disqualification: lack of sufficient arterial supply to the distal limb with occlusion of the radial and ulnar arteries. Only a tortuous interosseous artery supplied the amputation site. (b) Venography showed complete failure to opacify dominant distal veins with intravenous contrast.

Angiographic findings that precluded patients from consideration were instances where there was diminished arterial supply or venous drainage to the remaining limb resulting either secondary to the initial injury or to the subsequent surgeries (Figure 3(a)). One individual with significantly abnormal arterial examinations underwent separate venography. Absence of dominant venous return from the remaining limb was considered an absolute contraindication for transplant candidacy and resulted in disqualification of this individual (Figure 3(b)).

### 3.3. Postsurgical Follow-Up

#### 3.3.1. Musculoskeletal Postoperative Surveillance

**Figure 4:**
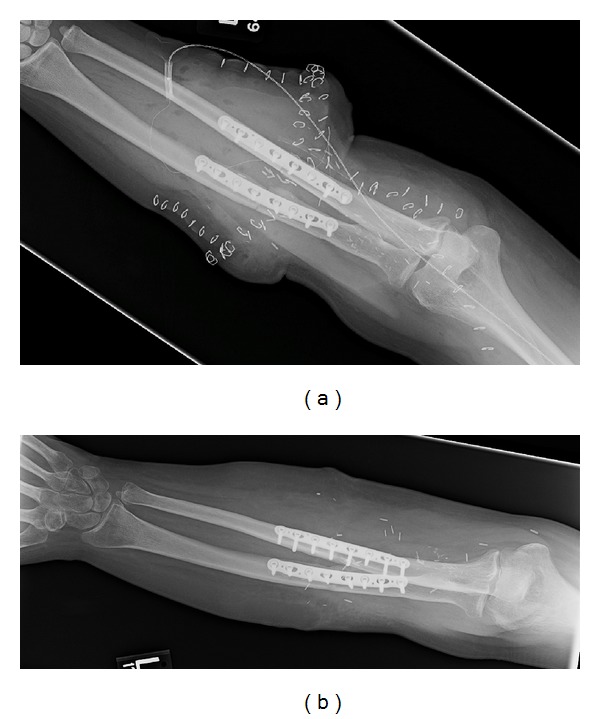
(a) Immediate postsurgical radiograph shows anatomic bony alignment and hardware with extensive soft tissue swelling. (b) One-year follow-up showing decreased swelling and interval osseous healing.

**Figure 5:**
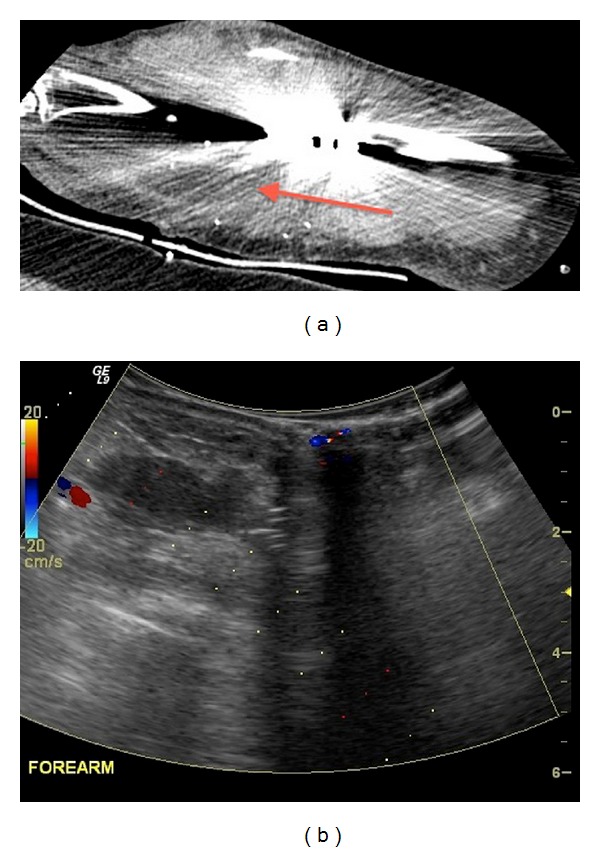
Postsurgical hematoma, questioned on CT and confirmed by ultrasound, as CT evaluation was significantly degraded by artifact from surgical hardware.

**Figure 6:**
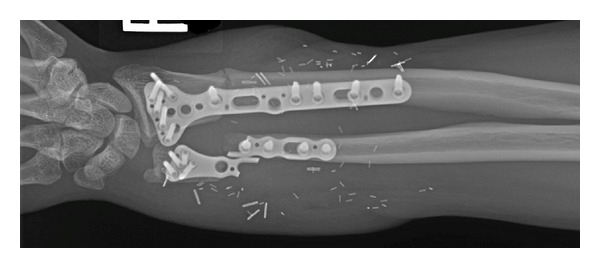
Radiograph showing development of nonunion and failed hardware.

Immediate postsurgical radiographs showed satisfactory osseous and hardware alignment in all patients. Follow-up radiographs obtained at 1, 3, 6, 9, and 12 months showed progressive osseous healing, maintained alignment, and diminishing soft tissue swelling (Figures 4(a) and 4(b)). Two patients developed postsurgical hematomas, one of which was detected on CT and confirmed by ultrasound, as CT evaluation was significantly degraded by artifact from surgical hardware (Figure 5). Follow-up imaging at 6 months and 1 year documented one episode of delayed-union that progressed to nonunion with failed hardware, prompting resection of the distal ulna and removal of the fractured fixation plate (Figure 6).

#### 3.3.2. Vascular Postoperative Surveillance

All five patients presented for routine surveillance with peripheral in-office ultrasound that was performed by the clinical service to check for signs of stenosis from endothelial proliferation as evidence of rejection. Postoperative angiography was performed at one year to reevaluate the vascular anastomoses. On CT angiography, one patient showed mild vascular narrowing at the anastomotic site without progressive narrowing on subsequent imaging. Given the stability, this was attributed to focal postoperative scarring rather than rejection. None of the patients progressed to the point of showing signs of rejection detectable by imaging, even when rejection was noticed clinically by skin biopsy. All transplants remained viable at the time of this submission with the exception of one patient who required explantationfollowing immunosuppression noncompliance. At the time of transplant removal, intraoperative angiography demonstrated patent vasculature. This was confirmed by peripheral sonography (12 MHz) at the level of the vascular anastomosis with normal velocities. However, due to the degree of skin thickening and edema, extensive beam attenuation limited the utility of ultrasound interrogation of the digital arteries.

## 4. Discussion

Extremity allotransplantation is immensely complex surgically, medically, and psychologically, necessitating life-long immunosuppression and compliance with intense physical rehabilitation. Furthermore, VCA remains a life-improving, rather than life-saving, procedure, requiring extensive clinical and imaging evaluation, as the level of tolerated morbidity is far less than for traditional life-saving organ transplantation. Additionally, the individuals considered for transplantation present with varying mechanisms of initial injury, a range of prior surgical repairs, and different degrees of physical, emotional, and psychological recovery. Thus, imaging is individually tailored as a set protocol may fail to appropriately characterize each candidate's medical and surgical past.

The selection of imaging modalities was affected by several factors. First, since each of these patients were committing to life-long surveillance imaging, much consideration was lent to limiting radiation exposure as much as possible. Due to the impact of long-term immunotherapy on renal function, attempts were made to limit total contrast dose when possible, with a preference given for conventional angiography over CT angiography. Another caveat with imaging selection was monetary, as all screening and subsequent imaging was provided for the patient. This partially accounted for the reliance on radiography and ultrasound over cross-sectional imaging with sinus radiographs and abdomen sonography (3–7 MHz) being used rather than CT during preoperative screening. Thus, imaging selection may vary between institutions depending on the investigational protocol in place.

### 4.1. Screening from a Musculoskeletal Point of View

Presurgical imaging was specifically performed to characterize the structural integrity of the native bones and soft tissues by identifying the level of healthy tissue and describing existing structural damage to guide the surgical approach. The goal of such imaging being to maximize the viability at the anastamotic site and the rehabilitation potential of the entire limb by ensuring adequate native soft tissues to support transplantation. Patients showing either arthropathy of the wrist or elbow were transplanted above the level of the diseased joint. Similarly, if the level of injury showed maceration of the distal residual tissues, with bone fragmentation or intra-articular fracture extension, transplantation would extend proximal to the level where muscle bulk and bone integrity were preserved. Thus, marked muscle atrophy of the proximal arm, significant rotator cuff or labral injury, and degenerative change of the shoulder were MRI findings that caused disqualification, as these features directly impacted the eventual functionality of the extremity that could not be bypassed surgically.

The limitation of preoperative assessment of the musculoskeletal system occurred in instances where the imaging findings did not correspond with the clinical performance of the patient. This was particularly evident in the instance of one individual with an unremarkable upper extremity MRI who failed consideration due to poor range-of-motion from extensive contractures not visible by imaging. This occasional discrepancy emphasized the importance of placing all imaging findings in the greater clinical context, as the radiographic review functioned as a component in the multifaceted preoperative assessment.

### 4.2. Screening from a Vascular Point of View

Both CT angiography and conventional angiography were used to evaluate vascular integrity and anatomy within the residual limb. Patients screened in 2008 typically underwent CT angiography while the later patients were screened using conventional angiography. This change was multifactorial, influenced in part by the increasing sensitivity to radiation exposure and awareness of renal drug sensitivity. In addition, technological advances facilitated the transition as the two modalities became fairly comparable in quality, but with the advantage that conventional imaging could be acquired using less contrast when performed by an experienced interventionalist [8–10].

At our institution, vascular mapping whether by CT or conventional angiography focused on identifying vessels adequate to serve as the vascular pedicle for the transplant. Features that defined acceptability were those proven in other transplant populations to decrease the degree of turbulence and thus complication, such as large vessel caliber, relative lack of branching, and maximum vessel length [8, 11–13]. Thus, a preference was placed on using the radial and ulnar arteries, rather than collaterals, and attention was directed to the length of the preserved vessels and their approximation to the distal-most aspect of the residual limb.

### 4.3. Musculoskeletal Postsurgical Surveillance

Since extremity composite tissue transplantation entails the transfer of multiple tissue types which each heal and reject independently, radiological surveillance attempted to monitor for signs of both processes [2, 3]. Postsurgical radiologic imaging focused on documenting transplant healing and the exclusion of postsurgical complication. These concerns were largely answerable by conventional radiography using serial radiographs, with CT and MRI being secondary modalities to further evaluate any irregularity detected on the initial radiograph or on physical examination. The structural concerns dictating imaging within the postsurgical period were akin to conventional orthopedic procedures with attention given to osseous alignment, progressive signs of healing, and surveillance for hardware failure.

It can be argued whether the radiologist should comment on the subjective bone density observed on follow-up radiographic studies given these patients' ongoing immunosuppressive therapy. Although little exists in the literature concerning the long-term effects of Tacrolimus (FK506) on bone healing in humans, FK506 has been shown to decrease trabecular bone density in rat models secondary to increased osseous resorption [14]. However, the effect on bone density is not well delineated with the newer immunosuppressive agents, even though decreased bone density is a known complication of extended corticosteroid use [15].

### 4.4. Postsurgical Surveillance from a Vascular Point of View

As with other transplant populations, surveillance of vascular patency was crucial both to avoid allograft failure and as an indication of possible rejection [11, 13]. Due to the portability and low associated risks associated with ultrasound, the majority of surveillance sonography was performed outside of the radiology department, unfortunately making the imaging unavailable for review and serving as a major limitation of this paper. Particularly as the referral to radiology for subsequent CT and conventional angiography occurred when abnormalities were reportedly detected by ultrasound that required further characterization.

In general terms, clinical ultrasound serves as a cost-effective surveillance tool. However, as with any dynamic medium, the modality is highly operator-dependent, with a theoretical risk of selection bias as only sample segments of the extremity vessels can be interrogated due to technical and time constraints [16, 17]. Thus, there exists the theoretical risk that a developing focal area of stenosis or occlusion may be missed, a risk minimized by correlation with angiography. Furthermore, as demonstrated by the single explantation, rejection at the skin and soft tissue levels can occur without affecting vessel patency due to the varying immunogenic potential of the different tissue types.

## 5. Conclusion

Vascular composite allotransplantation refers to a host of complex procedures entailing extensive detailed surgical planning to maximize returning form and functionality while simultaneously minimizing possible postsurgical complication. Both aims rely heavily on imaging to delineate the surgical approach and required donor tissue. Thus, preoperative imaging was used primarily to assess the remaining extremity for surgical planning and to exclude any underlying malady that could compromise transplant function or contraindicate life-long immunosuppression. Postoperative imaging served to evaluate transplant healing, identify any postsurgical complication and monitor for any indication of rejection. Thus, as VCA techniques become more common, the need for an understanding of how to appropriately image these patients will only increase in importance. It is the authors' hope that this paper inspires further clinical conversation that may translate to advances in future patient care.

## Figures and Tables

**Table 1:** Presurgical imaging evaluation in the accepted patients.

Modality	Purpose
Extremity radiograph (All)	Osseous integrity
CT (2)	Level of amputation
	Arthropathy
Angiography CT (3)	Vascular architecture
Conventional (4)*	Arterial and venous patency
HIP MRI/ABD US/ENT Radiographs (All)	Detection of underlying disease

Postoperative imaging	
Radiography (All) 1, 3, 6, 9, and 12 months	Bone healing/alignment
Peripheral US (All)	Vascular patency
Q 3 mo for year 1	Intimal hyperplasia
Angiography CT (3)	Vascular patency
Conventional (2)	

*Numbers total >5 because of bilateral evaluations.

**Table 2:** Summary of findings as detected on imaging.

Findings in residual limb	Systemic findings
Poor arterial supply (1)	Joint AVN (1)
Poor venous drainage (1)	Systemic disease (2)
Proximal arthropathy (1)	Inflammation/infection (1)
Extensive osteopenia (1)	
Insufficient soft tissues (3)	

## Advances in Knowledge

(1) Vascularized composite allotransplantation is intended to restore both form and function following catastrophic injury. (2) Such a complex surgical and clinical process depends on a spectrum of radiologic techniques to determine initial patient selection, surgical planning, and surveillance. (3) Musculoskeletal and vascular imaging findings may preclude transplant candidacy. (4) Special consideration must be given to this particular patient population, as they are committed to life-long imaging to monitor transplant viability.

## Implications for Patient Care

(1) Imaging plays a large role in determining transplant candidacy. (2) Radiologic findings may dictate whether or not a patient will be considered for allotransplantation and subsequent immunotherapy. (3) Thus, radiologists must be aware of the clinical implications of radiologic observations in this select patient population in order to maximize communication with the primary healthcare providers to ensure comprehensive patient care.

## Summary Statement

A discussion of imaging utilization for vascular composite allograft transplantation evaluation based on our institutional experience with hand allotransplantation.
